# Pyrazino[2,3-*b*]indolizine-10-carbonitrile

**DOI:** 10.1107/S1600536809008939

**Published:** 2009-03-14

**Authors:** Anita Stefańska, Dorota Zarzeczańska, Tadeusz Ossowski, Artur Sikorski

**Affiliations:** a University of Gdańsk, Faculty of Chemistry, Sobieskiego 18/19, 80-952 Gdańsk, Poland

## Abstract

In the crystal structure of the title compound, C_11_H_6_N_4_, neighbouring mol­ecules are linked into inversion dimers through pairs of weak C—H⋯N hydrogen bonds, forming an *R*_2_^2^(10) ring motif. The dimers forming this motif are further linked by π–π inter­actions. With respective average deviations from planarity of 0.004 (2) and 0.004 (1) Å, the pyrazino[2,3-β]indolizine and cyano fragment are oriented at 0.8 (1)° to each other. The mean planes of the pyrazino[2,3-*b*]indolizine skeleton either lie parallel or are inclined at an angle of 28.7 (2)° in the crystal.

## Related literature

For applications of this class of compounds, see: Akiyama *et al.* (1978 ▶); Foks *et al.* (2005 ▶); Kaliszan *et al.* (1985 ▶); Kushner *et al.* (1952 ▶); Mussinan *et al.* (1973 ▶); Petrusewicz *et al.* (1993 ▶, 1995 ▶); Seitz *et al.* (2002 ▶). For the synthesis, see: Pilarski & Foks (1981 ▶ and 1982 ▶). For the analysis of inter­molecular inter­actions, see: Spek (2009 ▶). For a description of the Cambridge Structural Database, see: Allen (2002 ▶). For hydrogen bonds, see: Steiner (1999 ▶).
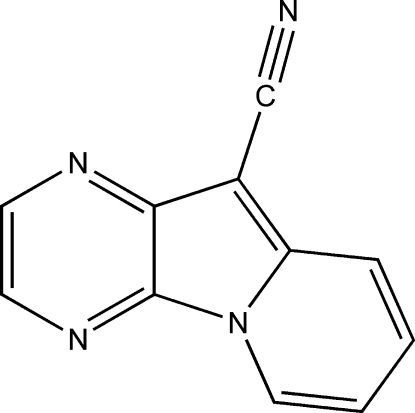


## Experimental

### 

#### Crystal data

C_11_H_6_N_4_
*M* *_r_* = 194.20
Monoclinic, 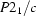

*a* = 3.8515 (5) Å
*b* = 14.147 (2) Å
*c* = 16.606 (3) Å
β = 91.260 (14)°
*V* = 904.6 (2) Å^3^
*Z* = 4
Mo *K*α radiation
μ = 0.09 mm^−1^
*T* = 295 K
0.30 × 0.08 × 0.06 mm

#### Data collection

Oxford Diffraction Ruby CCD diffractometer
Absorption correction: multi-scan (*CrysAlis RED*; Oxford Diffraction, 2008 ▶) *T*_min_ = 0.992, *T*_max_ = 0.999
6832 measured reflections
1606 independent reflections
1186 reflections with *I* > 2σ(*I*)
*R*_int_ = 0.032

#### Refinement

*R*[*F* ^2^ > 2σ(*F* ^2^)] = 0.040
*wR*(*F* ^2^) = 0.111
*S* = 1.02
1606 reflections
137 parameters
H-atom parameters constrained
Δρ_max_ = 0.18 e Å^−3^
Δρ_min_ = −0.15 e Å^−3^

### 

Data collection: *CrysAlis CCD* (Oxford Diffraction, 2008 ▶); cell refinement: *CrysAlis RED* (Oxford Diffraction, 2008 ▶); data reduction: *CrysAlis RED*; program(s) used to solve structure: *SHELXS97* (Sheldrick, 2008 ▶); program(s) used to refine structure: *SHELXL97* (Sheldrick, 2008 ▶); molecular graphics: *ORTEPII* (Johnson, 1976 ▶); software used to prepare material for publication: *SHELXL97* and *PLATON* (Spek, 2009 ▶).

## Supplementary Material

Crystal structure: contains datablocks I, global. DOI: 10.1107/S1600536809008939/ww2144sup1.cif

Structure factors: contains datablocks I. DOI: 10.1107/S1600536809008939/ww2144Isup2.hkl

Additional supplementary materials:  crystallographic information; 3D view; checkCIF report

## Figures and Tables

**Table 1:** Hydrogen-bond geometry (Å, °)

*D*—H⋯*A*	*D*—H	H⋯*A*	*D*⋯*A*	*D*—H⋯*A*
C2—H2⋯N12^i^	0.93	2.61	3.487 (2)	157

Symmetry code: (i) 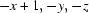.

**Table 2:** π–π interactions (Å, °)

*CgI*	*CgJ*	*Cg*⋯*Cg*	Dihedral angle	Interplanar distance	Offset
*A*	*B*^ii^	3.608 (1)	0.6	3.358 (1)	1.320 (1)

Symmetry codes: (ii) 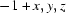. Notes: *CgA* and *CgB* are the centroids of the N1/C6–C8/C13 and N1/C2–C6 rings, respectively. The dihedral angle is that between the planes of the rings *CgI* and *CgJ*. The interplanar distance is the perpendicular distance of *CgI* from ring *J*. The offset is the perpendicular distance of ring *I* from ring *J*.

**Fig. 1.**
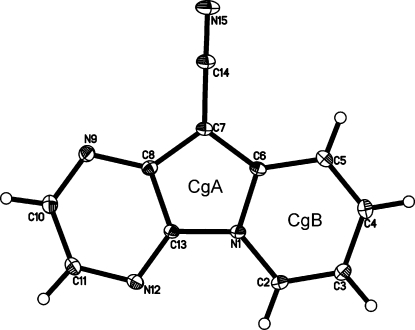
The molecular structure of the title compound showing the atom-labeling scheme. Displacement ellipsoids are drawn at the 25% probability level and H atoms are shown as small spheres of arbitrary radius. CgA and CgB denote the ring centroids.

**Fig. 2.**
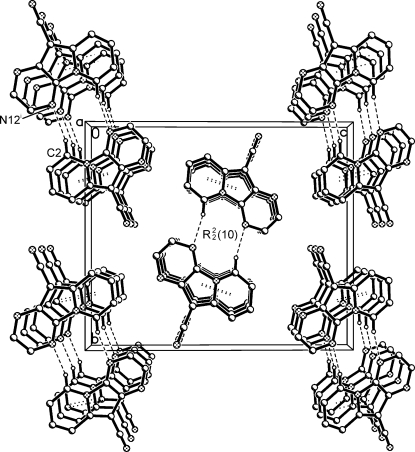
The arrangement of the molecules in the crystal structure viewed approximately along a axis. The C—H···N interactions are represented by dashed lines and the π–π interactions are represented by dotted lines. H atoms not involved in the interactions have been omitted. [Symmetry codes: (i) 1 - x, - y, - z; (ii) -1 + x, y, z.]

## supplementary crystallographic information

### Comment

Pyrazines play an important role as building block of many pharmaceutical products. They occur in many compounds with pharmaceutical and flavoring applications. Many of them have been found in nature. Pyrazines are responsible for flavour in foodstuffs, e.g. cheese, tea, coffee or cooked meat. (Akiyama *et al.*, 1978; Mussinan *et al.*, 1973). Biological activities of pyrazine derivatives are widely discussed in plentiful scientific publications: antibacterial (Foks *et al.*, 2005), anti-inflammatory (Petrusewicz *et al.*, 1995), chemotherapeutic agent (Kushner *et al.*, 1952), antimycobacterial (Seitz *et al.*, 2002) and antithrombotic (Petrusewicz *et al.*, 1993). Such pharmacological activities in group of pyrazine are possibly the result of their structures. It is known that the pyrazine-acetonitrile shows antiplatelet and analgestic activity (Kaliszan *et al.*, 1985). X-Ray structure of pyrazino[2,3-β]indolizine-10-carbonitrile is subject of the present paper.

In the Cambridge Structural Database (CSD; Version 5.27; Allen, 2002), there are no crystal structures containing the pyrazino[2,3-β]indolizine skeleton.

With average deviations from planarity of 0.004 (2) and 0.004 (1)Å respectively, the pyrazino[2,3-β]indolizine and cyano fragments are oriented at 0.8 (1)° to each other. The mean planes of the pyrazino[2,3-β]indolizine skeleton lie either parallel to or are inclined at an angle of 28.7 (2)° in the lattice.

In the crystal structure, neighbouring molecules are linked through weak C–H···N hydrogen bond forming R_2_^2^(10) ring motif (Table 1 and Fig. 2). Molecules which forming this motif are linked by π–π interactions between the central ring A and the lateral rings B (Table 2 and Fig. 2). All the interactions demonstrated were found by *PLATON* (Spek, 2009).

### Experimental

Pyrazino[2,3-β]indolizine-10-carbonitrile was obtained by mixing 2,3-dichloropyrazine, 2-pyridylacetonitrile and potassium carbonate in DMSO. The mixture was stirred for 5 h at 333 K. After cooling the reaction mixture to room temperature, water was added. Then mixture was acidified with hydrochloric acid (Pilarski & Foks, 1981 and 1982). The orange-green precipitate was obtained. Single crystals suitable for X-ray analysis were grown in methanol solution (m. p. = 486 K).

### Refinement

All H atoms were positioned geometrically and refined using a riding model, with C–H = 0.93Å and *U*_iso_(H) = 1.2*U*_eq_(C).

### Crystal data

**Table:**

C_11_H_6_N_4_	*F*(000) = 400
*M**_r_* = 194.20	*D*_x_ = 1.426 Mg m^−^^3^
Monoclinic, *P*2_1_/*c*	Mo *K*α radiation, λ = 0.71073 Å
Hall symbol: -P 2ybc	Cell parameters from 6832 reflections
*a* = 3.8515 (5) Å	θ = 3.0–25.0°
*b* = 14.147 (2) Å	µ = 0.09 mm^−^^1^
*c* = 16.606 (3) Å	*T* = 295 K
β = 91.260 (14)°	Needle, orange-green
*V* = 904.6 (2) Å^3^	0.30 × 0.08 × 0.06 mm
*Z* = 4	

### Data collection

**Table:**

Oxford Diffraction Ruby CCD diffractometer	1606 independent reflections
Radiation source: Enhance (Mo) X-ray Source	1186 reflections with *I* > 2σ(*I*)
graphite	*R*_int_ = 0.032
Detector resolution: 10.4002 pixels mm^-1^	θ_max_ = 25.1°, θ_min_ = 3.1°
ω scans	*h* = −4→4
Absorption correction: multi-scan (*CrysAlis RED*; Oxford Diffraction, 2008)	*k* = −16→15
*T*_min_ = 0.992, *T*_max_ = 0.999	*l* = −18→19
6832 measured reflections	

### Refinement

**Table:**

Refinement on *F*^2^	Secondary atom site location: difference Fourier map
Least-squares matrix: full	Hydrogen site location: inferred from neighbouring sites
*R*[*F*^2^ > 2σ(*F*^2^)] = 0.040	H-atom parameters constrained
*wR*(*F*^2^) = 0.111	*w* = 1/[σ^2^(*F*_o_^2^) + (0.0745*P*)^2^] where *P* = (*F*_o_^2^ + 2*F*_c_^2^)/3
*S* = 1.02	(Δ/σ)_max_ < 0.001
1606 reflections	Δρ_max_ = 0.18 e Å^−^^3^
137 parameters	Δρ_min_ = −0.15 e Å^−^^3^
0 restraints	Extinction correction: *SHELXL97* (Sheldrick, 2008), Fc^*^=kFc[1+0.001xFc^2^λ^3^/sin(2θ)]^-1/4^
Primary atom site location: structure-invariant direct methods	Extinction coefficient: 0.017 (5)

### Special details

**Table:**

Experimental. Empirical absorption correction using spherical harmonics, implemented in SCALE3 ABSPACK scaling algorithm.
Geometry. All esds (except the esd in the dihedral angle between two l.s. planes) are estimated using the full covariance matrix. The cell esds are taken into account individually in the estimation of esds in distances, angles and torsion angles; correlations between esds in cell parameters are only used when they are defined by crystal symmetry. An approximate (isotropic) treatment of cell esds is used for estimating esds involving l.s. planes.
Refinement. Refinement of *F*^2^ against ALL reflections. The weighted *R*-factor *wR* and goodness of fit *S* are based on *F*^2^, conventional *R*-factors *R* are based on *F*, with *F* set to zero for negative *F*^2^. The threshold expression of *F*^2^ > σ(*F*^2^) is used only for calculating *R*-factors(gt) *etc*. and is not relevant to the choice of reflections for refinement. *R*-factors based on *F*^2^ are statistically about twice as large as those based on *F*, and *R*- factors based on ALL data will be even larger.

### Fractional atomic coordinates and isotropic or equivalent isotropic displacement parameters (Å^2^)

**Table:**

	*x*	*y*	*z*	*U*_iso_*/*U*_eq_	
N1	0.1906 (3)	0.18749 (8)	0.01142 (7)	0.0395 (3)	
C2	0.3590 (4)	0.15549 (12)	−0.05644 (9)	0.0463 (4)	
H2	0.4296	0.0928	−0.0597	0.056*	
C3	0.4196 (4)	0.21534 (13)	−0.11734 (10)	0.0541 (5)	
H3	0.5303	0.1943	−0.1632	0.065*	
C4	0.3135 (4)	0.31070 (13)	−0.11094 (11)	0.0554 (5)	
H4	0.3574	0.3523	−0.1529	0.066*	
C5	0.1475 (4)	0.34299 (12)	−0.04437 (10)	0.0506 (4)	
H5	0.0804	0.4060	−0.0415	0.061*	
C6	0.0772 (4)	0.28097 (10)	0.02016 (9)	0.0412 (4)	
C7	−0.0876 (4)	0.29055 (10)	0.09485 (9)	0.0431 (4)	
C8	−0.0708 (4)	0.20124 (11)	0.13408 (9)	0.0410 (4)	
N9	−0.1882 (3)	0.17236 (10)	0.20656 (8)	0.0500 (4)	
C10	−0.1199 (4)	0.08189 (13)	0.22182 (10)	0.0538 (5)	
H10	−0.1906	0.0569	0.2706	0.065*	
C11	0.0522 (4)	0.02209 (12)	0.16866 (10)	0.0528 (4)	
H11	0.0890	−0.0403	0.1844	0.063*	
N12	0.1684 (3)	0.04892 (9)	0.09592 (8)	0.0482 (4)	
C13	0.1002 (3)	0.13879 (10)	0.08115 (9)	0.0387 (4)	
C14	−0.2351 (4)	0.37523 (12)	0.12502 (10)	0.0528 (5)	
N15	−0.3567 (4)	0.44383 (12)	0.14836 (11)	0.0782 (5)	

### Atomic displacement parameters (Å^2^)

**Table:**

	*U*^11^	*U*^22^	*U*^33^	*U*^12^	*U*^13^	*U*^23^
N1	0.0429 (6)	0.0342 (7)	0.0413 (7)	0.0010 (5)	−0.0001 (5)	−0.0047 (5)
C2	0.0487 (8)	0.0447 (9)	0.0455 (9)	0.0043 (7)	0.0020 (7)	−0.0093 (7)
C3	0.0561 (10)	0.0600 (12)	0.0466 (10)	0.0024 (8)	0.0074 (8)	−0.0039 (8)
C4	0.0558 (9)	0.0571 (11)	0.0533 (11)	−0.0042 (8)	0.0013 (8)	0.0129 (8)
C5	0.0533 (9)	0.0398 (9)	0.0586 (11)	−0.0007 (7)	−0.0023 (8)	0.0044 (8)
C6	0.0401 (7)	0.0331 (8)	0.0501 (10)	−0.0016 (6)	−0.0033 (7)	−0.0045 (7)
C7	0.0463 (8)	0.0351 (9)	0.0479 (9)	0.0010 (6)	0.0007 (7)	−0.0075 (7)
C8	0.0407 (8)	0.0406 (9)	0.0415 (9)	−0.0042 (6)	−0.0013 (6)	−0.0059 (7)
N9	0.0535 (7)	0.0499 (10)	0.0467 (9)	−0.0028 (6)	0.0035 (6)	−0.0017 (6)
C10	0.0568 (9)	0.0560 (12)	0.0485 (10)	−0.0065 (8)	0.0020 (8)	0.0058 (8)
C11	0.0607 (9)	0.0430 (10)	0.0543 (10)	−0.0034 (8)	−0.0049 (8)	0.0086 (8)
N12	0.0545 (7)	0.0373 (8)	0.0525 (8)	0.0030 (6)	−0.0043 (6)	−0.0006 (6)
C13	0.0407 (7)	0.0339 (9)	0.0413 (9)	−0.0012 (6)	−0.0026 (6)	−0.0032 (6)
C14	0.0552 (9)	0.0436 (11)	0.0597 (11)	0.0000 (8)	0.0016 (8)	−0.0123 (8)
N15	0.0841 (11)	0.0517 (11)	0.0992 (13)	0.0092 (8)	0.0080 (10)	−0.0274 (9)

### Geometric parameters (Å, °)

**Table:**

N1—C2	1.3883 (19)	C7—C14	1.422 (2)
N1—C13	1.3980 (18)	C7—C8	1.422 (2)
N1—C6	1.4014 (18)	C8—N9	1.3580 (19)
C2—C3	1.343 (2)	C8—C13	1.419 (2)
C2—H2	0.9300	N9—C10	1.330 (2)
C3—C4	1.414 (3)	C10—C11	1.400 (2)
C3—H3	0.9300	C10—H10	0.9300
C4—C5	1.368 (2)	C11—N12	1.352 (2)
C4—H4	0.9300	C11—H11	0.9300
C5—C6	1.416 (2)	N12—C13	1.3201 (19)
C5—H5	0.9300	C14—N15	1.149 (2)
C6—C7	1.412 (2)		
			
C2—N1—C13	129.87 (13)	C6—C7—C14	125.53 (14)
C2—N1—C6	122.96 (13)	C6—C7—C8	107.47 (12)
C13—N1—C6	107.18 (11)	C14—C7—C8	126.98 (15)
C3—C2—N1	119.85 (15)	N9—C8—C13	122.01 (14)
C3—C2—H2	120.1	N9—C8—C7	131.34 (14)
N1—C2—H2	120.1	C13—C8—C7	106.65 (13)
C2—C3—C4	119.29 (15)	C10—N9—C8	112.96 (13)
C2—C3—H3	120.4	N9—C10—C11	123.77 (15)
C4—C3—H3	120.4	N9—C10—H10	118.1
C5—C4—C3	121.36 (16)	C11—C10—H10	118.1
C5—C4—H4	119.3	N12—C11—C10	124.36 (15)
C3—C4—H4	119.3	N12—C11—H11	117.8
C4—C5—C6	120.35 (16)	C10—C11—H11	117.8
C4—C5—H5	119.8	C13—N12—C11	111.60 (13)
C6—C5—H5	119.8	N12—C13—N1	125.19 (13)
N1—C6—C7	109.19 (12)	N12—C13—C8	125.30 (14)
N1—C6—C5	116.18 (13)	N1—C13—C8	109.50 (13)
C7—C6—C5	134.63 (14)	N15—C14—C7	179.0 (2)
			
C13—N1—C2—C3	179.88 (14)	C6—C7—C8—C13	−0.94 (16)
C6—N1—C2—C3	0.1 (2)	C14—C7—C8—C13	−179.67 (14)
N1—C2—C3—C4	0.6 (2)	C13—C8—N9—C10	1.07 (19)
C2—C3—C4—C5	−0.6 (2)	C7—C8—N9—C10	−179.86 (16)
C3—C4—C5—C6	−0.1 (2)	C8—N9—C10—C11	−0.5 (2)
C2—N1—C6—C7	179.16 (12)	N9—C10—C11—N12	−0.1 (3)
C13—N1—C6—C7	−0.67 (14)	C10—C11—N12—C13	0.2 (2)
C2—N1—C6—C5	−0.79 (19)	C11—N12—C13—N1	179.34 (12)
C13—N1—C6—C5	179.38 (12)	C11—N12—C13—C8	0.4 (2)
C4—C5—C6—N1	0.8 (2)	C2—N1—C13—N12	1.2 (2)
C4—C5—C6—C7	−179.16 (15)	C6—N1—C13—N12	−178.99 (13)
N1—C6—C7—C14	179.76 (14)	C2—N1—C13—C8	−179.75 (12)
C5—C6—C7—C14	−0.3 (3)	C6—N1—C13—C8	0.07 (14)
N1—C6—C7—C8	1.01 (15)	N9—C8—C13—N12	−1.1 (2)
C5—C6—C7—C8	−179.06 (15)	C7—C8—C13—N12	179.60 (14)
C6—C7—C8—N9	179.88 (14)	N9—C8—C13—N1	179.82 (11)
C14—C7—C8—N9	1.2 (3)	C7—C8—C13—N1	0.55 (15)

### Hydrogen-bond geometry (Å, °)

**Table:**

*D*—H···*A*	*D*—H	H···*A*	*D*···*A*	*D*—H···*A*
C2—H2···N12^i^	0.93	2.61	3.487 (2)	157

Symmetry codes: (i) −*x*+1, −*y*, −*z*.

### Table 2 π–π interactions (Å, °)

**Table:**

*CgI*	*CgJ*	*Cg*···*Cg*	Dihedral angle	Interplanar distance	Offset
*A*	*B*^ii^	3.608 (1)	0.6	3.358 (1)	1.320 (1)

Symmetry codes: (ii) -1 + *x*, *y*, *z*. Notes: *CgA* and *CgB* are the centroids of the N1/C6–C8/C13 and N1/C2–C6 rings, respectively. The dihedral angle is that between the planes of the rings *CgI* and *CgJ*. The interplanar distance is the perpendicular distance of *CgI* from ring *J*. The offset is the perpendicular distance of ring *I* from ring *J*.
